# Supplementary material for: A VLP vaccine platform comprising the core protein of hepatitis B virus with N-terminal antigen capture
Source: Int J Biol Macromol. Author manuscript; Available in PMC 2025 Aug 14. (PMC7618004; doi:10.1016/j.ijbiomac.2025.141152)
Supplement: supporting data, tables and figures [file EMS207520-supplement-supporting_data__tables_and_figures.pdf]

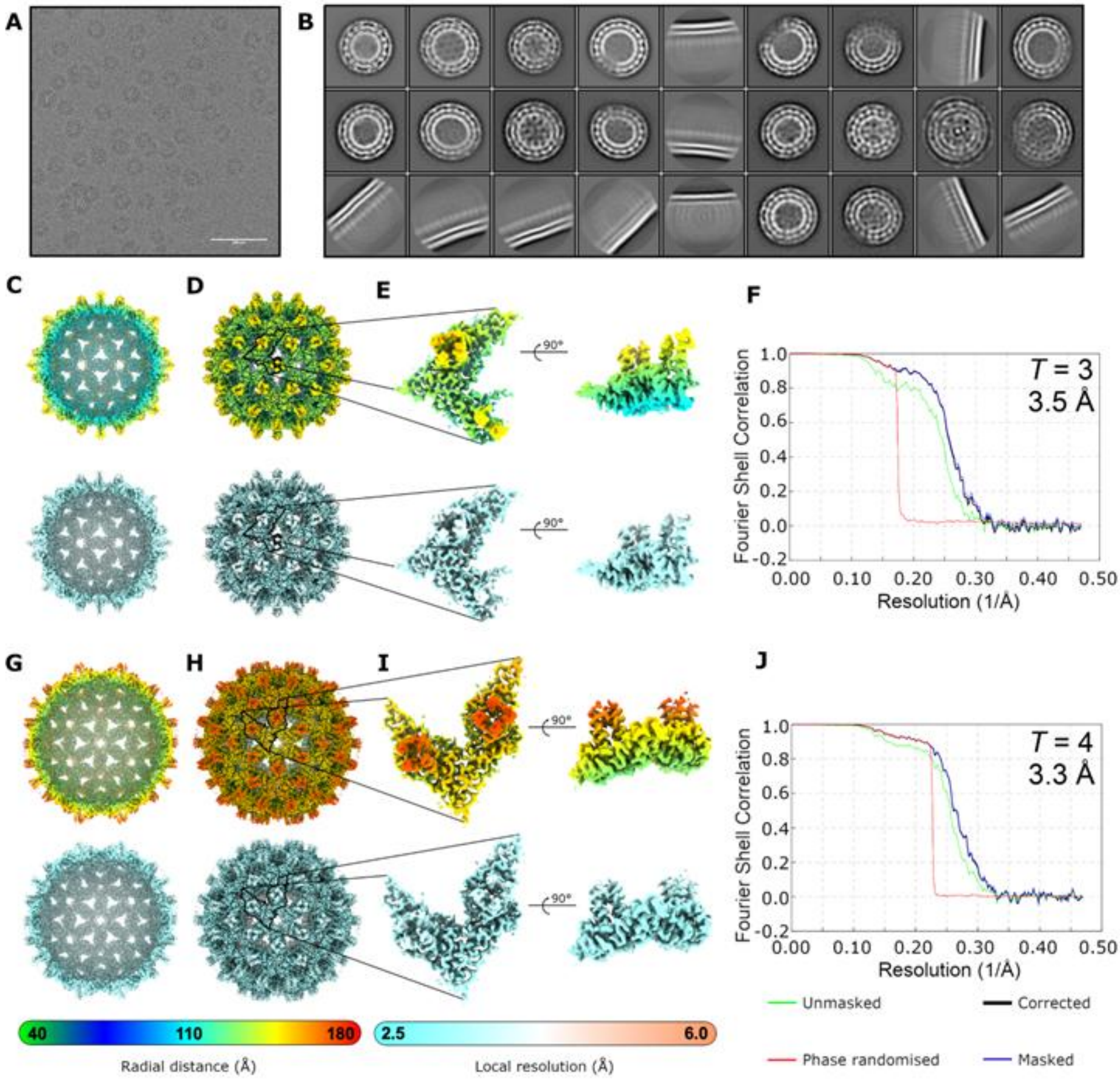

Figure S1. Processing of N-VelcroVax cryo-EM dataset. (A) Representative micrograph from N-VelcroVax data collection. Scale bar shows 100 nm. (B) 2D classes containing the most particles following automated particle picking. Data were two-fold down-sampled prior to classification. (C-E) Density map for *T* = 3 VLP of N-VelcroVax filtered according to local resolution, including (C) central section (D) whole VLP and (E) enlarged views of the asymmetric unit. (F) FSC plot for *T* = 3 N-VelcroVax reconstruction. (G-I) Density map for *T* = 4 VLP of N-VelcroVax. (J) FSC plot for *T* = 4 N-VelcroVax reconstruction. All reconstructions shown at ~4 σ and coloured according to radial distance or local resolution, as indicated.

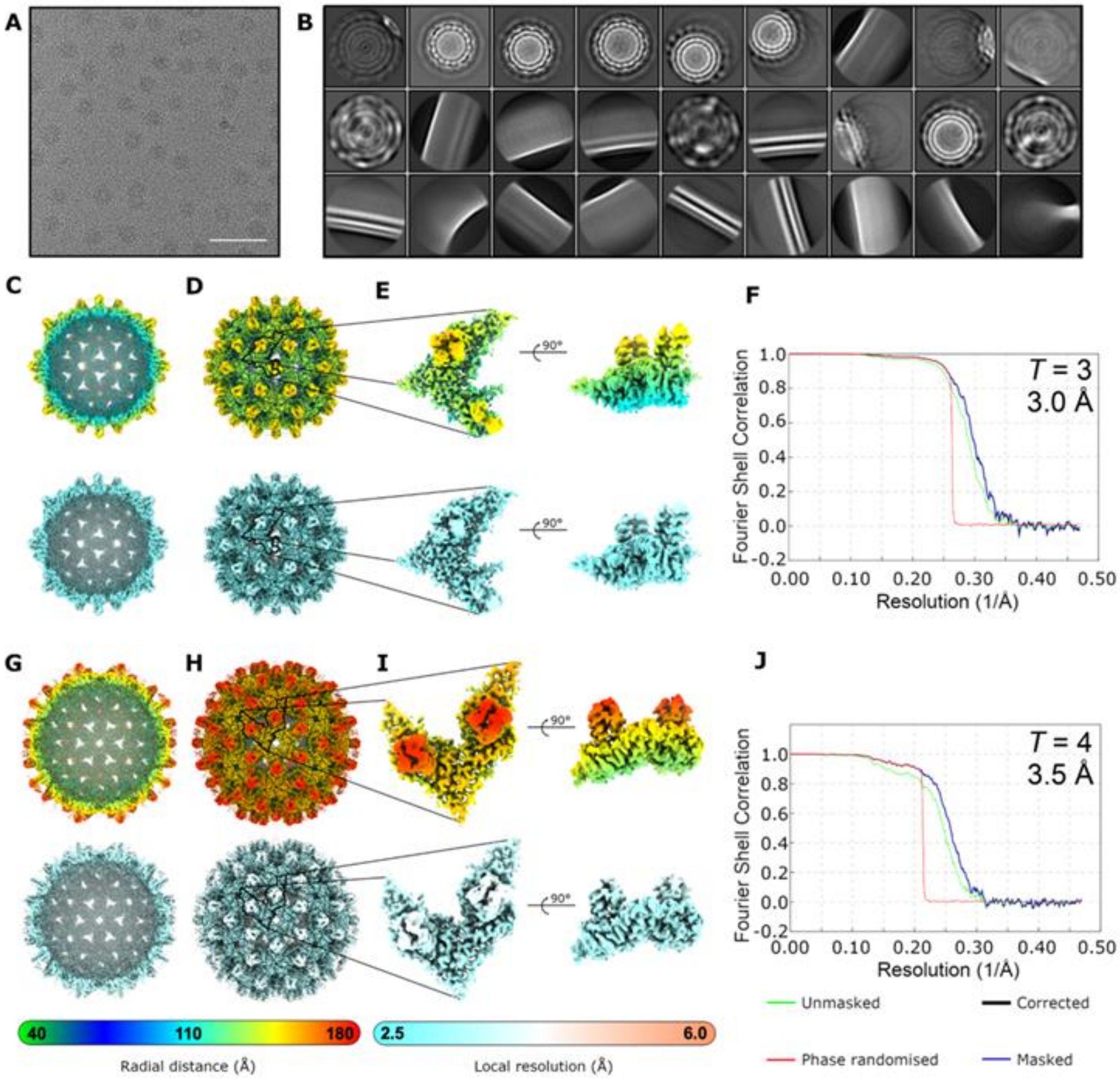

Figure S2. Processing of N-VelcroVax:SUMO-gp1 cryo-EM dataset. (A) Representative micrograph from N-VelcroVax:SUMO-gp1 data collection. Scale bar shows 100 nm. **(B)** 2D classes containing the most particles following automated particle picking. Data was five-fold down-sampled prior to classification. **(C-E)** Density map for T = 3 VLP filtered according to local resolution, including **(C)** central section **(D)** whole VLP and **(E)** enlarged views of the asymmetric unit. **(F)** FSC plot for T = 3 N-VelcroVax:SUMO-gp1 reconstruction. **(G-I)** Density map for T = 4 VLP. **(J)** FSC plot for T = 4 N-VelcroVax:SUMO-gp1 reconstruction. All reconstructions shown at ~3 σ and coloured according to radial distance or local resolution, as indicated.

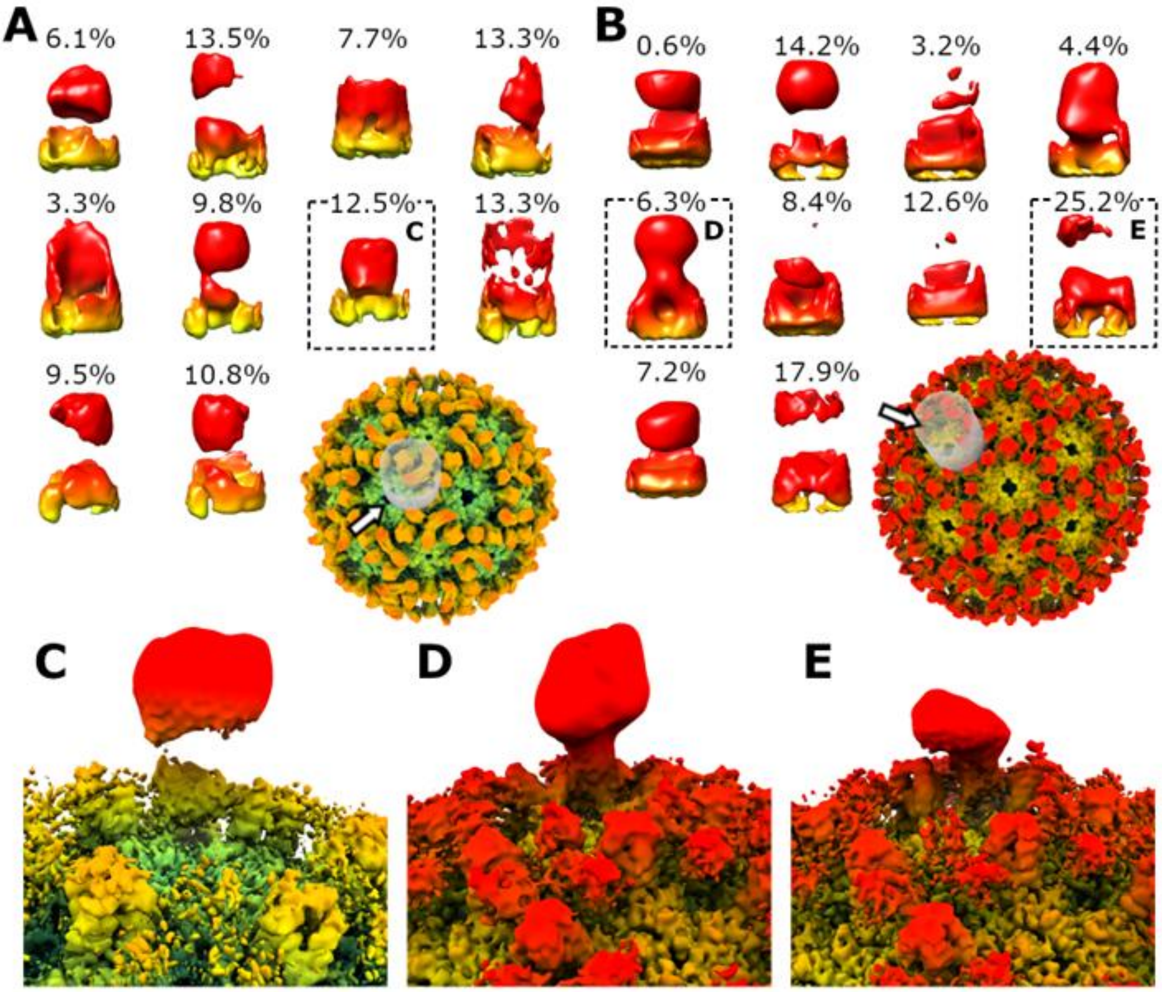

Figure S3. Focussed classification of N-VelcroVax:SUMO-gp1 cryo-EM dataset. (A, B) Density observed in all 10 focussed classes from focussed classification of **(A)** *T* = 3 N-VelcroVax:SUMO-gp1 and **(B)** *T* = 4 N-VelcroVax:SUMO-gp1. The number above each class indicates the proportion of sub-particles that were assigned. The position of the mask (grey) is shown for reference. Classes are shown oriented from the viewpoint indicated by the white arrows. **(C-E)** Asymmetric reconstructions using particles contained in the focussed classes indicated by dashed boxes in (A) and (B), filtered by local resolution.

Table S1: N-VelcroVax and N-VelcroVax:SUMO-Junín gp1 cryo-EM data collection and processing parameters.

Sample	N-VelcroVax		N-VelcroVax:SUMO-gp1	
Microscope	FEI Titan Krios		FEI Titan Krios	
Detector mode	Linear		Linear	
Camera	Falcon III		Falcon IV	
Voltage (kV)	300		300	
Pixel size (Å)	1.065		1.065	
Nominal magnification	75,000×		75,000×	
Exposure time (s)	1.0		1.3	
Total dose (e^-/Å^2)	43		54.7	
Number of fractions	30		40	
Defocus range (µm)	–0.5 to –2.9		–0.5 to –2.9	
Number of micrographs	12,797		23,966	
Acquisition software	Thermo Scientific EPU		Thermo Scientific EPU	
	T = 3	T = 4	T = 3	T = 4
EMDB ID	EMD-50832	EMD-50833	EMD-50834	EMD-50835
PDB ID	PDB-9FWE	PDB-9FWF	N/A	N/A
Number of particles contributing to map	40,254	56,416	298,802	57,537
Map resolution (FSC = 0.143) (Å)	3.5	3.3	3.0	3.5
Map resolution range around atom positions (Å)	3.2 – 4.2	3.1 – 5.4	2.8 – 3.6	3.3 – 6.0
Map sharpening B factor (Å^2)	-187	-179	-203	-223
