# Supplementary material for: A VLP vaccine platform comprising the core protein of hepatitis B virus with N-terminal antigen capture
Source: Int J Biol Macromol. Author manuscript; Available in PMC 2025 Aug 14. (PMC7618004; doi:10.1016/j.ijbiomac.2025.141152)
Supplement: Appendix [file EMS207520-supplement-Appendix.pdf]

Appendix A. Supplementary data

Supplementary data to this article can be found online at https://doi.org/10.1016/j.ijbiomac.2025.141152.
